# Microplastics (Polystyrene) Exposure Induces Metabolic Changes in the Liver of Rare Minnow (*Gobiocypris rarus*)

**DOI:** 10.3390/molecules27030584

**Published:** 2022-01-18

**Authors:** Chunling Wang, Miaomiao Hou, Kunyu Shang, Huanshan Wang, Jianwei Wang

**Affiliations:** 1 The Key Laboratory of Aquatic Biodiversity and Conservation of Chinese Academy of Sciences, Institute of Hydrobiology, Chinese Academy of Sciences, Wuhan 430072, China; chwang@ihb.ac.cn (C.W.); houmiao006@163.com (M.H.); hswang@ihb.ac.cn (H.W.); 2 University of Chinese Academy of Sciences, Beijing 100049, China; 3 College of Fisheries, Huazhong Agricultural University, Wuhan 430070, China; 15127315124@163.com

**Keywords:** plastic pollution, fishes, oxidative stress, lipid metabolism, energy metabolism

## Abstract

Microplastics are environmental contaminants and an emergent concern. Microplastics are abundant in freshwater and can cause biochemical stress in freshwater organisms. In the current study, rare minnows (*Gobiocypris rarus*) were exposed to 1μm polystyrene microplastics at 200 μg/L concentration. We observed various sublethal effects after four weeks of exposure but no mortality. Numerous cellular and tissue alterations were observed in the liver. Differential metabolites and differentially expressed genes between control and exposure groups were identified and mapped to pathways in the Kyoto Encyclopedia of Genes and Genomes. The combination of transcriptomic and metabolomic analyses revealed significantly varied metabolic pathways between the two groups. These pathways were involved in glucolipid, amino acid, and nucleotide metabolism. Results demonstrated that MP exposure induced immune reaction, oxidative stress, and disturbed glycolipid and energy metabolism. The current study provided novel insights into the molecular and metabolic mechanisms of microplastic ecotoxicology in rare minnow.

## 1. Introduction

Plastics are widely used for industrial production and daily life because of their advantageous features, such as water resistance, durability, light weight, insulation, and corrosion resistance. In 2018, global plastic production reached approximately 360 million tons, of which 80,000 tons are estimated to seep into the aquatic environment [1]. Each year, 12.7 million tons of plastic debris enter the ocean, and plastic constitutes the world’s largest marine debris [2]. Plastics that enter the environment can be broken down into smaller particles or fragments through physical, chemical, and biological processes. The tiny plastic fragments directly discharged into the environment lead to severe microplastics (MPs) pollution [3]. Microplastics are 100 nm^–5^ sized plastic beads or fragments, as recognized by most authors [4,5]. MPs are ubiquitous in all ecosystems [6,7]. MPs are similar to plankton in shape and size and therefore, are eaten by fish frequently. As a result, various MPs accumulate in freshwater and marine fish species [8,9,10]. Several studies have revealed that microplastics can be ingested by many aquatic organisms, such as copepods, nematodes, brown shrimp, crabs, and mussels [11,12,13].

Microplastics, a new type of pollutants, have received significant attention from scholars and the public globally. The study of microplastics pollution has become one of the current international research hotspots [14,15,16,17,18]. Recently, research reports on the sources, types, distribution, and toxicological effects of microplastics on aquatic organisms have been increasing [1,19,20]. Microplastics can accumulate in aquatic organisms, endangering organisms through physical damage, carrier effects (plasticizer release, enrichment effect with other pollutants), bioaccumulation, and food chain transmission [21,22,23]. MPs might induce toxic effects at the individual level (grazing, reproduction, survival, growth) [24], tissue level (inflammatory response, oxidative damage, fatty vacuoles), cellular level (hepatocyte necrosis) [25], and genetic level (variety of endocrine disrupting gene expression) [26,27]. However, only a few reports about the toxic effects of microplastics on fish are present, including *Danio rerio*, *Oryzias latipes*, and *Dicentrarchus labrax* [25,28]. Therefore, there is a need to obtain more fish toxicology data, analyze the mechanism, and accurately evaluate the ecological risk of microplastics for the environment.

Rare minnow (*Gobiocypris rarus*), the most standardized model fish in China, is suitable for chemical testing and ecotoxicological research [29,30]. However, toxic effects of microplastics on rare minnows are not well discussed. Polystyrene (PS) is produced industrially and distributed in freshwater bodies in China [31]. In our previous study, we observed that MPs entered the fish body through the digestive tract and damaged the gut and liver. Additionally, several studies have reported the effects of 1 μm MPs on organisms [32,33,34]. Therefore, in the present study, rare minnows (*Gobiocypris rarus*) were exposed to 200 μg/L of 1 μm fluorescent polystyrene (PS) particles, for 28 days, and multiple endpoints including histopathology, transcriptome, and metabolome were analyzed.

The primary objective of the current study was to study the effects of polystyrene microplastics on rare minnow liver after exposure for 28 days, particularly in metabolic pathways, by using a combination of metabolomic, transcriptomic data, and histopathological analysis.

## 2. Results

### 2.1. Condition during This Study

During the experiment, the MPs concentration of the test solution was (3.71 ± 0.10) × 10^8^ items/L, and the dissolved oxygen, conductivity, pH, and temperature of the control and MPs-treated groups were in the normal range (Table 1). Throughout the study, fish remained in excellent condition.

### 2.2. Histopathological Changes Induced by PS-MPs Treatment

**Figure 1:**
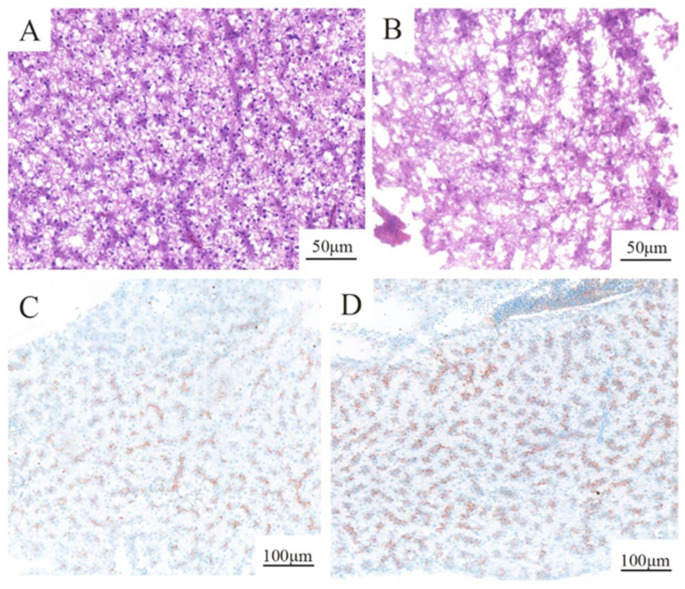
Liver tissues of rare minnow. (**A**,**C**) Normal appearance of the control liver. (**B**) The liver was exposed to 200 μg/L MPs solution showing cell hypertrophy and increased vacuolization in hepatocytes compared with the control. (**D**) The liver exposed to 200 μg/L MPs shows a larger area and daker color of the lipid droplets than the control.

Cytoplasmic vacuolization and irregularity were observed in the liver tissues of MPs-treated fish. Compared with the control (Figure 1A,C), the exposure group exhibited cell hypertrophy and increased vacuolization of hepatocytes (Figure 1B) after H&E staining. After staining with oil red O, the lipid droplets in the liver increased in the exposure group, and a larger area and darker color of the lipid droplets were easily observed (Figure 1D). However, no partials were observed in the liver in the present study (Figure S1).

### 2.3. Transcriptome Analysis

**Figure 2:**
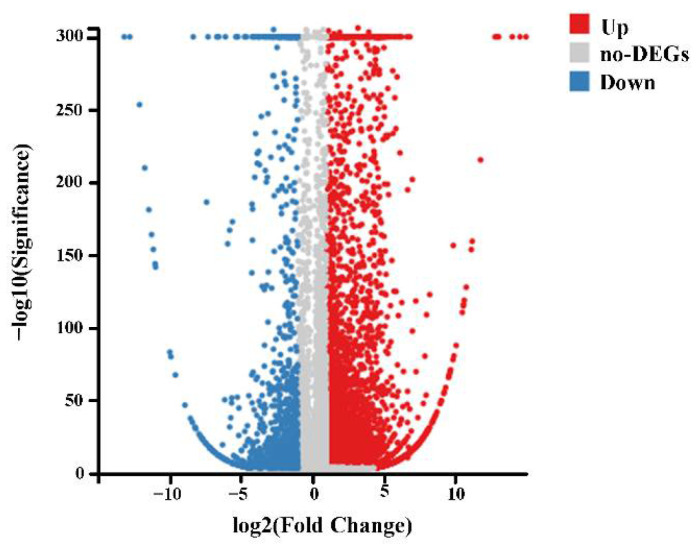
Volcano plot of DEGs. Upregulated genes are shown as red dots, whereas downregulated genes are shown as blue dots.

**Figure 3:**
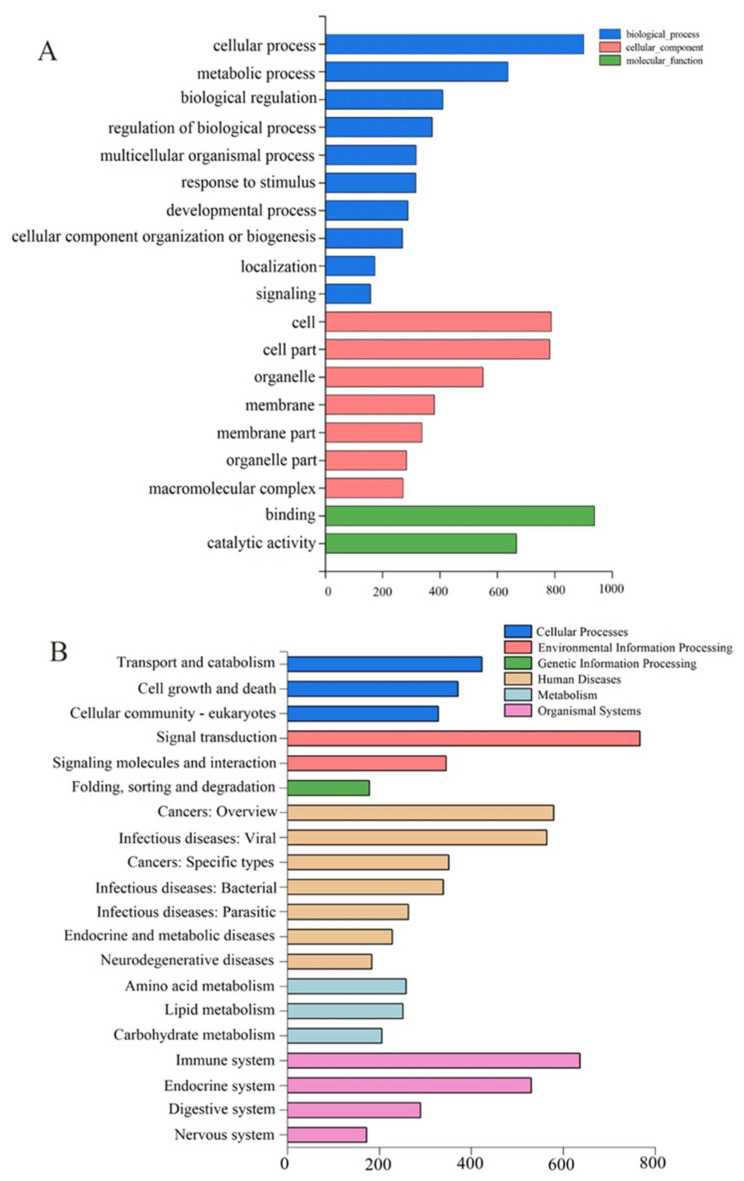
Transcriptome analysis of rare minnows exposed to 200 μg/L MPs compared with the control group. (**A**) GO enrichment analysis. (**B**) KEGG pathway analysis.

We obtained 71.69, 71.7, 71.7, 71.79, 71.6, and 71.67 million 150 bp sequence reads from six samples of the control and MPs-treated groups. Among these, 6882 upregulated (red dots) and 1276 downregulated (blue dots) differentially expressed genes (DEGs) were observed between control and MPs-treated groups (change fold > 2, Q-value ≤ 0.001) (Figure 2). Gene Ontology (GO) enrichment analysis was carried out to demonstrate biological function of DEGs. Enriched GO terms comprised three categories: biological process, cellular component, and molecular function. The most significantly enriched terms were as follows: “cellular process”, “metabolic process”, “binding”, “catalytic activity”, “cell”, and “cell part” (Figure 3A). Pathway analyses were conducted using the Kyoto Encyclopedia of Genes and Genomes (KEGG) database to characterize the potential pathways involving DEGs functionally. The results revealed a diverse range of pathways, with 14,587 DEGs between control and exposure groups assigned to 341 pathways. KEGG enrichment analysis demonstrated a significant enrichment of “signal transduction”, “immune system”, “transport and catabolism”, “endocrine system”, and “infectious diseases” (Figure 3B). Moreover, genes with the highest differential expression (top 20 genes) are listed in Table 2. These top 20 genes were related to response to various biological processes including histidine metabolism, immune process, glucuronidation process, cancer tumorigenesis, cell division, inflammatory reaction, cell growth, cell death, differentiation, genetic information, environmental information process, and metabolism process.

### 2.4. Metabolomic Analysis

The results of the principal component analysis (PCA) did not distinguish the control group from the exposure group (Figure S2a,b). Orthogonal partial least squares discriminant analysis (OPLS-DA) was also performed to evaluate group separation, and the comparison between control and exposure is shown in Figure S2c,d. The results indicated that the OPLS-DA models were reliable, which revealed a clear separation between control and exposure groups (Figure S2e,f). Then, the differential metabolites (DMs) of the liver were detected using non-targeted analyses, and the results illustrated that 41 metabolites (26 DMs in ESI+ and 15 DMs in ESI-) were dysregulated in control compared with the exposure group. These metabolites were tentatively identified based on comparing their accurate experimental molecular mass values with the corresponding molecular mass values reported in various online databases (mass error of the possible molecular formulas, 10 ppm). Table 3 shows the list of tentatively identified metabolites, differentiating between the control and MPs-exposed samples in ESI+ and ESI- mode, respectively. Metabolite identity, ions detected, molecular mass, relative mass error, *p*-value, fold-change, folding trend, and KEGG C-code are included in the table. several DMs were involved in glycolipid and pyrimidine metabolism.

**Figure 4:**
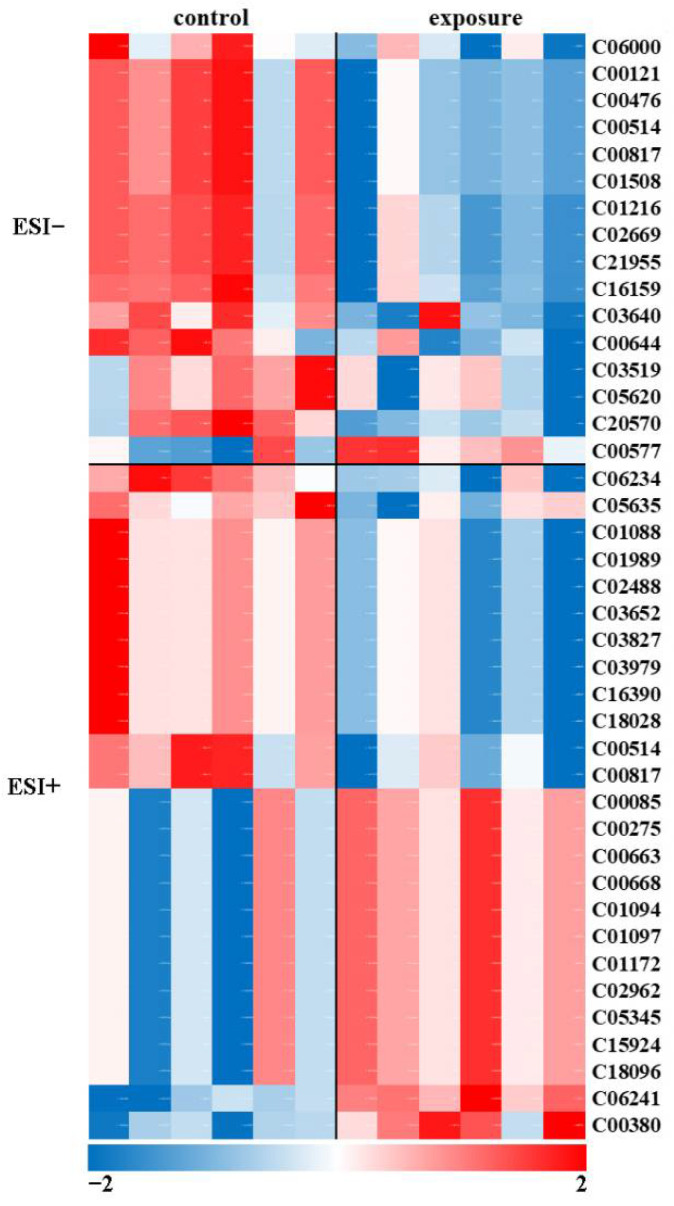
Metabolomic analysis of rare minnows exposed to 200 μg/L MPs compared with the control group.

The hierarchical clustering heat map reveals similar patterns of metabolite change in each group, revealing slight variations between biological replicates possibly due to natural biological variations. In Figure 4, the relative change in the metabolite concentrations is depicted with colors in the heatmap. Increased and decreased metabolites are represented in red and blue colors, respectively. Hierarchical clustering of metabolites revealed two primary clusters, with one including metabolites whose concentrations increased upon MPs exposure and the other including metabolites that were depleted by the treatment. Furthermore, the annotated KEGG pathways were classified according to the KEGG type (Figure S3). KEGG pathway analysis illustrated that many DMs were present in “pentose and glucuronate interconversions”, “fructose and mannose metabolism”, and “carbon metabolism” (Figure S3).

### 2.5. Confirmation of DEGs by qRT-PCR

**Figure 5:**
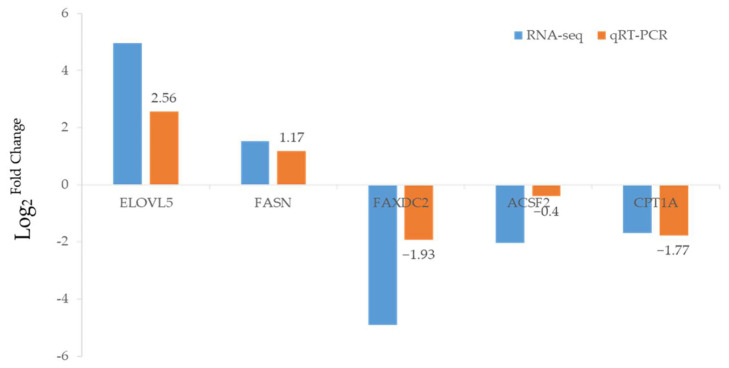
Comparisons of the expression profiles of 5 DEGs obtained using RNA-seq and qRT-PCR analysis. The expression levels of selected genes were normalized to β-actin rRNA.

To confirm the RNA-seq data, 5 DEGs involved in “lipid metabolism” were selected for qRT-PCR validation. These genes included carnitine O-palmitoyl transferase 1(CPT1A), fatty-acyl-CoA synthase (ACSF2), fatty acid hydroxylase do-main-containing protein 2 (FAXDC2), elongation of very long-chain fatty acids protein 5 (ELOVL5), and fatty acid synthase, animal type (FASN). The results revealed that gene expression patterns of the two methods were consistent (Figure 5), indicating the specificity and accuracy of the transcriptome expression analysis.

### 2.6. Pathway-Based Integration of Metabolomic and Transcriptomic Datasets

**Figure 6:**
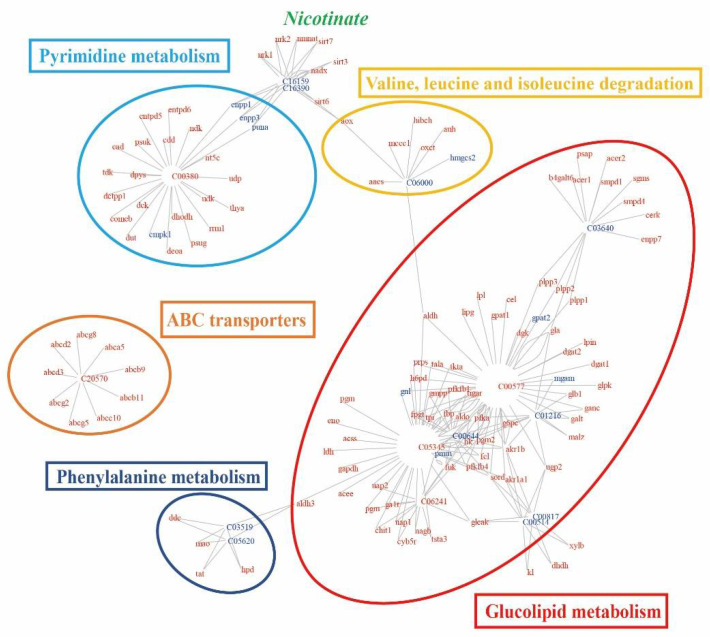
Integrated network of functional interactions between metabolites (represented by their KEGG C-codes) and genes (represented by their abbreviation names) whose levels were affected in rare minnow by MP exposure. Metabolites and genes are connected if they share at least one common KEGG pathway. Increased or decreased metabolite or mRNA abundances (genes) in MP-exposed samples are indicated by red and blue symbols, respectively. Standard names of some relevant metabolites are shown in green.

Integration of metabolomic and transcriptomic datasets improves the understanding of potential biological processes to gain insight into their mechanisms. Shared KEGG pathways between the control and exposure groups were identified. We chose 123 genes and 16 metabolites (Table S1) to construct the bipartite graph for visualization, shown in Figure 6. Genes and metabolites exhibited diverse colors with increased (red symbols) and decreased (blue symbols) mean. KEGG pathway analysis of the aggregated metabolite and gene biomarkers identified 14 functional modules related to metabolism, including more than three biomarkers (Table S1). All modules included at least three genes and one metabolite. Among 16 metabolites, two drew the most attention: d-Mannitol 1-phosphate (C05345) and d-Glyceraldehyde (C00577), which involved five different pathways individually. Additionally, 27 genes were involved in at least two functional modules [35].

The largest cluster of biomarkers comprised metabolites and genes related to glucolipid metabolism (red oval in Figure 6). The cluster was constructed by glycometabolism (fructose and mannose metabolism, galactose metabolism, amino sugar and nucleotide sugar metabolism, pentose and glucuronate interconversions, glycolysis/gluconeogenesis, and pentose phosphate pathway) and lipid-related metabolism (glycerolipid metabolism and sphingolipid metabolism). A second intensively populated cluster included biomarkers related to pyrimidine metabolism (blue oval in Figure 6). Additionally, valine, leucine and isoleucine degradation, ABC transporters, and phenylalanine metabolism are shown in the diagram.

## 3. Discussion

### 3.1. MP Exposure Induced Immune Reaction and Oxidative Stress in Rare Minnow

In the present study, transcriptomics, metabolomics, and histology results indicate early symptoms of liver damage. Cell hypertrophy and vacuolization in hepatocytes were observed in MPs-treated fish, indicating early inflammation in the liver. Moreover, similar results occurred through metabolomic and transcriptomic analysis. “Immune system” and “infectious diseases” pathways were enriched in transcriptomic analysis. Complement component 3 (C3) and component 4 (C4) were included in the top 20 changed genes. Complement was a crucial component of the innate immune system. Complement fragments play a vital role in inflammatory reactions, complex immune clearance, and antibody production. C3 and C4 expression changes indicate immune response in rare minnow. C3 and C4 exhibited different expression patterns in the present study, as complement is a highly sophisticated defense system [36]. Similar results have been reported in zebrafish. After exposure for 3 weeks to 2000 µg/L PS-MPs (5 µm and 70 nm diameter), early inflammatory responses, necrosis, and infiltration were observed in hepatocytes of zebrafish [25]. After feeding with PVC and PE for 21 days, histopathological damage was detected in the intestine and liver, with altered immune parameters in European sea bass *Dicentrarchus labrax* [28]. In addition, inflammatory responses were observed in mussels treated with PS and PE MPs [37]. MPs are recognized as foreign substances and stimulate or suppress fish immune responses by inducing immune-toxicity, meaning that MPs can affect fish immunity through various mechanisms [38].

**Figure 7:**
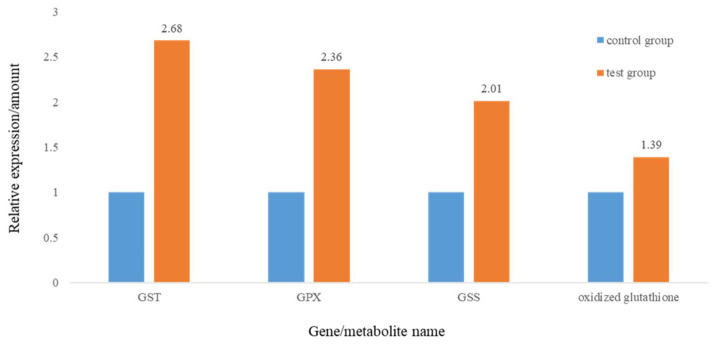
Relative changes of genes and metabolite involved in glutathione metabolism.

In addition, oxidative stress caused by the overproduction of reactive oxygen species (ROS) is one of the most commonly measured biomarkers for environmental contaminant exposure, including MP exposure [39,40,41], consistent with our results. In our study, the relative content of oxidized glutathione (GSSG) was increased. Furthermore, the expression of glutathione S-transferase, glutathione peroxidase, and glutathione synthase was increased (Figure 7). Glutathione, a crucial cellular antioxidant, plays a crucial role in preventing oxidative damage and the toxicity of xenobiotic electrophiles in organisms [42,43]. MPs could induce intracellular ROS levels and cause inflammation and oxidative stress [23,37,44]. Exposure to MPs influences ROS production in rare minnows, stimulating antioxidant reactions and disturbing glutathione and its dependent cycle reactions [38].

### 3.2. MP Exposure Disturbed Glycolipid and Energy Metabolism in Rare Minnow

MPs exposure also disturbed the metabolomic profiles in fish liver, providing additional insights into the molecular mechanisms of toxicity induced by MPs. These altered metabolites were involved in carbohydrates, fatty acids, amino acids, and nucleic acid and appeared as a primary response to MPs exposure. Significant perturbation occurred in most monosaccharide metabolism pathways, including galactose metabolism, fructose and mannose metabolism, pentose phosphate pathway, pentose and glucuronate interconversions, and glycolysis/gluconeogenesis. Energy production from these metabolic pathways was also disturbed. For instance, d-glyceraldehyde (C00577) and beta-d-Fructose 6-phosphate (C05345) contents, which play a vital role in the pentose phosphate pathway, were increased in the current research. In general, pentose phosphate pathway functions include the production of sugar phosphates as intermediates for biosynthesis and NADPH, a biological reducing agent, together with several secondary function dependent metabolites [45]. Additionally, glycolysis/gluconeogenesis are the primary pathways related to energy metabolism. Hence, MP exposure triggered energy metabolism change and disturbed material supply to a wide range of biological processes, including lipid, amino acid, and carbohydrate synthesis [46].

MP exposure induced abnormal lipid metabolism in rare minnows, including glycerolipid and sphingolipid metabolism. Pathway-based integration analysis revealed that the key metabolite in lipid metabolism (C00577) concentration increased. The alteration can be correlated with the changes in gene expression in the same pathway, such as alcohol dehydrogenase, aldehyde reductase, glycerol kinase, and diacylglycerol O-acyltransferase, which confirmed the alteration in lipid metabolism. Additionally, lipid droplets, which plays a vital role in intracellular lipid storage and lipid metabolism regulation, were numerous and larger in the exposed group liver [47]. Our result was consistent with the metabonomic results. Combining expression patterns of genes related to lipid synthesis (elovl5, fasn) with lipolysis (faxdc2, acsf2, cat1a), it was determined that lipogenesis was triggered and lipolysis was inhibited during lipid accumulation.

In conclusion, MP exposure may trigger lipid synthesis and inhibit lipolysis in rare minnow. Lu et al. (2016) discovered that microplastics could alter the metabolic profile in adult zebrafish liver and disturb lipid and energy metabolism [28]. Wan et al. (2019) also demonstrated that 5/50 mm PS-MP exposure altered the genes associated with glycolysis/lipid metabolism in the larval zebrafish, which proved the effects of PS-MP on disturbing the metabolism of energy and glycolipids [39]. Additionally, microplastics induce intestinal lipid metabolism disorders in zebrafish, and six-lipid metabolism-related metabolites of propylene glycol, linoleic acid, palmitic acid, carnitine, triglycerides, and Trimethylamine N-oxide (TMAO) were significantly altered in MPs-treated zebrafish guts [48]. These results are in accordance with our study. The current study investigated the histology and metabolic changes of lipid and revealed perturbation of lipid storage and lipid metabolism in rare minnow. Lipid metabolism is an essential cellular process that converts nutrients into metabolic intermediates for membrane biosynthesis, energy storage, and signaling molecule generation [49,50]. Furthermore, lipids play a vital role in reproduction and sexual maturation, immunological responses, and environmental adaptation [51]. Therefore, MP exposure might disturb several biological processes in rare minnows.

In addition to the largest cluster, we observed changes in biochemical pathways related to amino acids and pyrimidine. Isoleucine, valine, and leucine are branched-chain amino acids (BCAAs), promoting fatty acid metabolism and preventing fat accumulation. Isoleucine, leucine, valine, and lysine can regulate energy metabolism [37], and reduction in these metabolites indicated that PS-MPs exposure induced lipid and energy metabolism disruption in rare minnow. Pyrimidines are the catabolic products of nucleotides and can be uptaken and reutilized by the cells of the other organs to support protein synthesis [52]. In rare minnow, relative metabolites and genes were increased, which may help produce a larger amount of material for subsequent biological processes.

## 4. Materials and Methods

### 4.1. Fish Maintenance

Mixed-sex rare minnows were obtained from the Institute of Hydrobiology, Chinese Academy of Science. The mean total length, body length, and wet weight (±standard deviation) of subadult fish (age, 3 months) were 31.18 ± 1.59 mm, 25.23 ± 1.20 mm, and 0.32 ± 0.07 g, respectively. The fish were acclimated in 8 L glass tanks for 7 days before the experiment and fed fresh Artemia nauplii twice daily. During the acclimation period, fish were maintained at 25 ± 1 °C and subjected to a photoperiod of 16:8 h (light/dark) [53]. The culture water in the tank was refreshed every 48 h. All experimental procedures were approved by the Animal Care and Use Committee of the Institute of Hydrobiology, Chinese Academy of Sciences (Approval Protocol No. Y913101101), and all the experiments were conducted in accordance with the guidelines of the committee.

### 4.2. MPs Exposure

The PS-MPs (1 μm diameter beads) labeled with green fluorescence (excitation wavelength, 488 nm; emission wavelength, 518 nm; *w*/*v* = 10 mg/mL; ρ = 1.05 g/cm^3^, C.V = 5–10%) were purchased from Tianjin Baseline ChromTech Research Center (Tianjin, China). The size of the particles was confirmed using a scanning electron microscope (SEM) (Figure S4). Before configuring the test solution, the PS-MPs were shaken for 30 min (40,000 Hz) with an ultrasonic instrument to produce a homogeneous mixture. Acclimated rare minnows were randomly assigned to 10 glass tanks (5 replicates each in the experiment and control group), and each tank contained 12 fish and 6 L test solution. The test solution was prepared for the treatment group by dispersing MPs in water to achieve a final MPs concentration of 200 μg/L (≈ 3.6 × 10^8^ items/L). The exposure concentration was chosen in view of the environmental concentration and other studies about the toxic effects of microplastics on aquatic organisms [54,55]. During the experiment, the test solution in each tank was refreshed every 48 h, and all tanks were continually aerated to maintain particles dispersion in water. Fish were exposed to the diluting water without MPs for the control group. All the other conditions were consistent with those in the acclimation period.

After exposure for 28 days, rare minnows were sampled on the same day, rinsed to remove the particles from the skin, and euthanized by general anesthesia. Depending on the experiment’s aim, the fish’s liver was dissected, immediately frozen in liquid nitrogen and stored at −80 °C for metabolomic analysis and transcriptomic analysis or fixed in 10% formalin for histopathological analysis.

### 4.3. Histopathological Analysis

Six fish from each treatment group were used to assess liver tissue damage. The tissues were fixed in 10% formalin, embedded in paraffin wax, sectioned at 4 μm thickness, and stained with eosin (H&E) and oil red O for observation of organizational form and lipid droplet precipitation, respectively. The scanned slices were histopathologically analyzed [56]. To observe the presence of fluorescent-labeled polystyrene microplastic particles in liver, one bright-field image was acquired by microscopy first, and then a dark-field image of the slide was acquired by epifluorescence microscopy [47].

### 4.4. RNA Extraction, Library Preparation, and Transcriptome Sequencing

We prepared six different sequencing libraries for RNA-seq as follows: Trizol Reagent (Invitrogen, Carlsbad, CA, USA) was utilized to extract total RNA from frozen samples of rare minnow according to the manufacturer’s protocol. Nine fish from each group were used for the transcriptome analysis. The livers of every three fish were pooled and used for extraction. All extracted samples were stored at −80 °C until analysis. Subsequently, total RNA was qualified and quantified using a Nano Drop and Agilent 2100 bioanalyzer (Thermo Fisher Scientific, Waltham, MA, USA) [40, 57].

Oligo(dT)-attached magnetic beads were used to purify mRNA. After addition of fragmentation buffer to generate short mRNA fragments (each of approximately 200 bp), random hexamer primers were applied to synthesize the first-strand cDNA. Buffer, dNTPs, RNase H, and DNA polymerase I were added to synthesize the second strand. The cDNA fragments were amplified by PCR, purified by Ampure XP Beads, then dissolved in EB solution. The double-stranded PCR products were heated, denatured, and circularized by the splint oligo sequence to obtain the final library. Each library was sequenced on the BGIseq500 platform (BGI-Shenzhen, China).

To gain insight to the change of phenotype, GO and KEGG enrichment analysis of annotated differentially expressed genes was performed by Phyper based on the hypergeometric test. The sequencing data were filtered with SOAPnuke (v1.5.2) [58], and clean reads were obtained and stored in FASTQ format. Essentially, differential expression analysis was performed using DESeq2 (v1.4.5) [59] with Q-value ≤ 0.05.

### 4.5. Metabolite Analysis

Liver samples were collected and stored at −80 °C until analysis. Metabolites were first extracted with 800 μL of pre-cooled precipitant (methanol/acetonitrile/pure water = 2:2:1). After grinding (60 Hz, for 4 min), samples were subjected to ultrasonic treatment (80 HZ, 10 min) and stored at −20 °C for 120 min. Then, the samples were centrifuged for 15 min (25,000 g, 4 °C), and the supernatant was obtained in a dryer. Then, 50 μL of each sample was mixed into a quality control sample. The raw data of the mass spectrometer were preprocessed in Xevo G2-XS QTOF (Waters, Manchester, UK), and peak extraction was performed primarily through Progenesis QI (version 2.2). Principal component analysis (PCA) and orthogonal partial least squares discriminant analysis (OPLS-DA) were performed to analyze the principal components of each group before screening the differential metabolites. Afterwards, the metabolites with VIP > 1 and *p* < 0.05 were selected as the differential metabolites (DMs). Finally, the DMs of positive and negative modes were combined to conduct KEGG pathway analysis.

### 4.6. Metabolomics and Transcriptomics Combination Analysis

To obtain a more comprehensive insight into the abnormally regulated pathways, pathway-based integration of metabolomic and transcriptomic datasets was performed with the reshape2 and igraph packages in R [60]. Additionally, the Pearson correlation coefficient (PCC) and the relevant *p*-value were evaluated to assess the correlation between the differential metabolites and DEGs. Any given metabolite was considered linked to a given gene if they shared at least one common KEGG pathway. Only PCC greater than 0.90 and Pearson correlation coefficient *p*-value (PCCP) less than 0.05 were considered significant.

### 4.7. Validation of DEGs by qRT-PCR

To validate the reliability of data obtained by RNA-seq, qRT-PCR was performed. Eighteen fish from each group were used, and five DEGs were randomly selected. Total RNA from the liver of each fish was isolated through an SV total RNA isolation system kit (Promega, Madison, WI, USA). Then, 4 μg of isolated total RNA was transcribed to cDNA through the RevertAid™ First Strand cDNA Synthesis Kit (Fermentas, Waltham, MA, USA) with oligo-dT primers. Next, qRT-PCR was performed using the Fast Start Universal SYBR Green Master Mix (Roche, Mannheim, Germany). Primer sequences are listed in Table S2. Three replicates were included for each sample, and the β-actin gene was used as an internal control for normalization of gene expression. Relative expression levels were measured in threshold cycle value and normalized using the equation 2^−ΔΔCt^ method [61].

### 4.8. Data Analyses

Histopathological analysis was performed using CaseViewer 2.2. We tested data normality and variance homogeneity. After that, a one-way ANOVA test followed by Dunnett’s test were used to evaluate the statistical differences of gene expression between the control and treatment group in IBM SPSS Statistics 20.

## 5. Conclusions

Non-targeted LC-MS metabolomics, RNA-seq transcriptomic and histology analyses reflected a defined metabolic disruption in rare minnow exposure to PS-MPs. These non-targeted approaches were used to identify a complete disruption compared with the usually used targeted techniques with limited profiling capability [35]. Integration of both transcriptomic and metabolomic results at the pathway level revealed the presence of affected metabolic routes in rare minnow. MP exposure may induce immune reaction and oxidative stress and disturb glycolipid and energy metabolism in rare minnow. Studies have demonstrated that lipids are susceptible to oxidation. Oxidative stress and inflammation could impair lipid metabolism [62]. MPs exposure may stimulate lipogenesis and inhibit lipolysis in rare minnow. Further investigation requires additional efforts. Our study provided new insights into the molecular and metabolic characteristics underlying rare minnow exposure to MP on a theoretical basis. However, the potential toxic effects are primarily unclear. More efforts are required in multivariate-based integration.

## Figures and Tables

**Table 1:** Physical and chemical characteristics of the test water.

Group	Before/After Refreshing Water	Dissolved Oxygen (mg/L)	Conductivity (μs/cm)	pH	Temperature (°C)
Control	before	7.55 ± 0.36	747 ± 7	8.03 ± 0.17	26.8 ± 1.1
	after	7.77 ± 0.15	732 ± 3	8.29 ± 0.06	27.3 ± 0.9
MPs-treated	before	7.57 ± 0.46	750 ± 9	8.04 ± 0.22	26.6 ± 1.2
	after	7.71 ± 0.20	734 ± 2	8.25 ± 0.07	27.1 ± 1.0

**Table 2:** The top 20 DEGs after MP exposure.

Gene Name	Description	Log2fc	Q-Value	Biological Process	Function
hutU	urocanate hydratase	−12.08	5.27 × 10^−254^	Histidine metabolism	Catalyzes the conversion of urocanic acid
C3	Complement component 3	−11.71	2.10 × 10^−210^	Immune process	Part of the complement system
UGT	glucuronosyltransferase	−11.43	1.25 × 10^−181^	Glucuronidation process	Making relative enzymes
C1R	Complement component 1, r subcomponent	−11.24	1.66 × 10^−164^	Immune process	Part of the complement system
MHC1	major histocompatibility complex	−10.96	3.80 × 10^−142^	Immune process	Making proteins in certain immune system cells
FKBP4	FK506-binding protein 4	−9.89	2.23 × 10^−80^	Immune process	Signal transduction
HSD11B2	corticosteroid 11-beta-dehydrogenase isozyme 2	−9.57	8.38 × 10^−68^	Immune process	Modulates intracellular glucocorticoid levels
FMN2	formin 2	−8.90	5.06 × 10^−47^	Organismal Systems	Development
RAP1GAP	RAP1 GTPase activating protein 1	9.60	8.43 × 10^−68^	Cancer tumorigenesis	GTPase activator
NRAS	GTPase NRas	9.67	1.35 × 10^−70^	Cell division	Signal transduction
RELA	transcription factor p65	9.69	2.28 × 10^−71^	Inflammatory reaction	Signal transduction
C4	Complement component 4	10.52	6.84 × 10^−111^	Immune process	Part of the complement system
TACC3	transforming acidic coiled-coil-containing protein 3	10.59	2.30 × 10^−115^	Cell growth and differentiation	Signal transduction
CAMK1	calcium/calmodulin- dependent protein kinase I	10.61	1.48 × 10^−116^	Cell growth and death	Signal transduction
TF	transferrin	10.65	2.84 × 10^−119^	Environmental Information Process	Signal transduction
A2M	alpha-2-macroglobulin	10.79	3.15 × 10^−128^	Immune process	Proteinase inhibitor
PLAUR	plasminogen activator, urokinase receptor	9.35	3.49 × 10^−59^	Immune process	Signal transduction
RNF19A	E3 ubiquitin-protein ligase RNF19A	9.25	3.81 × 10^−56^	Genetic Information Process	Translation
SHOC2	leucine-rich repeat protein SHOC2	9.04	6.74 × 10^−50^	Environmental Information Process	Signal transduction
ALDH	aldehyde dehydrogenase	8.74	2.74 × 10^−42^	Metabolism process	Producing aldehyde enzymes

**Table 3:** List of statistically significant tentatively identified metabolites to differentiate between control and MPs-exposed rare minnow liver samples.

Compound	Molecular Formula	Mode	Measured Molecular Mass (Da)	Mass Error (ppm)	Fold-Change	Trend	*p*-Value	Kegg C-Code
2-Formylglutarate	C6H8O5	ESI-	159.0293	−3.91076	0.64	down	0.002	C16159
2-Dehydro-3-deoxy-d-galactonate	C6H10O6	ESI-	177.04	−2.53718	0.63	down	0.001	C01216
d-Galactono-1,5-lactone	C6H10O6	ESI-	177.04	−2.53718	0.63	down	0.001	C02669
l-Galactono-1,5-lactone	C6H10O6	ESI-	177.04	−2.53718	0.63	down	0.001	C21955
d-Mannitol 1-phosphate	C6H15O9P	ESI-	307.043	−2.00474	0.67	down	0.023	C00644
d-Altronate	C6H12O7	ESI-	195.0505	−2.85294	0.61	down	0.001	C00817
l-Lyxose	C5H10O5	ESI-	195.0505	−3.72763	0.61	down	0.001	C01508
d-Ribose	C5H10O5	ESI-	195.0505	−3.72763	0.61	down	0.001	C00121
d-Lyxose	C5H10O5	ESI-	195.0505	−3.72763	0.61	down	0.001	C00476
d-Mannonate	C6H12O7	ESI-	195.0505	−2.85294	0.61	down	0.001	C00514
(S)-3-Hydroxyisobutyryl-CoA	C25H42N7O18P3S	ESI-	852.1423	−2.79862	0.59	down	0.044	C06000
Sphingosyl-phosphocholine	C23H50N2O5P+	ESI-	510.3457	3.888599	0.64	down	0.040	C03640
N-Acetyl-d-phenylalanine	C11H13NO3	ESI-	252.087	−3.40067	0.68	down	0.048	C05620
N-Acetyl-l-phenylalanine	C11H13NO3	ESI-	252.087	−3.40067	0.68	down	0.048	C03519
d-Mannonate	C6H12O7	ESI+	219.0459	−8.34259	0.67	down	0.010	C00514
d-Altronate	C6H12O7	ESI+	219.0459	−8.34259	0.67	down	0.010	C00817
5-Hydroxyindoleacetate	C10H9NO3	ESI+	192.0659	1.905397	0.61	down	0.044	C05635
(R)-3,3-Dimethylmalate	C6H10O5	ESI+	180.0877	6.414412	0.66	down	0.016	C01088
l-Fucono-1,5-lactone	C6H10O5	ESI+	180.0877	6.414412	0.66	down	0.016	C18028
(S)-2-(Hydroxymethyl)glutarate	C6H10O5	ESI+	180.0877	6.414412	0.66	down	0.016	C16390
2-Dehydro-3-deoxy-l-fuconate	C6H10O5	ESI+	180.0877	6.414412	0.66	down	0.016	C03827
3-Ethylmalate	C6H10O5	ESI+	180.0877	6.414412	0.66	down	0.016	C01989
(2R,3S)-2,3-Dimethylmalate	C6H10O5	ESI+	180.0877	6.414412	0.66	down	0.016	C03652
(R)-2-Ethylmalate	C6H10O5	ESI+	180.0877	6.414412	0.66	down	0.016	C02488
2-Dehydro-3-deoxy-l-rhamnonate	C6H10O5	ESI+	180.0877	6.414412	0.66	down	0.016	C03979
4-Methyl-l-glutamate	C6H11NO4	ESI+	144.0655	-0.08917	0.48	down	0.006	C06234
alpha-1,5-l-Arabinobiose	C10H18O9	ESI-	281.0882	1.31699	0.78	down	0.004	C20570
beta-d-Glucose 1-phosphate	C6H13O9P	ESI+	283.019	0.420669	1.28	up	0.046	C00663
beta-d-Fructose 6-phosphate	C6H13O9P	ESI+	283.019	0.420669	1.28	up	0.046	C05345
alpha-d-Glucose 6-phosphate	C6H13O9P	ESI+	283.019	0.420669	1.28	up	0.046	C00668
d-Mannose 6-phosphate	C6H13O9P	ESI+	283.019	0.420669	1.28	up	0.046	C00275
d-Tagatose 6-phosphate	C6H13O9P	ESI+	283.019	0.420669	1.28	up	0.046	C01097
d-Fructose 1-phosphate	C6H13O9P	ESI+	283.019	0.420669	1.28	up	0.046	C01094
d-Allulose 6-phosphate	C6H13O9P	ESI+	283.019	0.420669	1.28	up	0.046	C18096
l-Gulose 1-phosphate	C6H13O9P	ESI+	283.019	0.420669	1.28	up	0.046	C15924
beta-d-Glucose 6-phosphate	C6H13O9P	ESI+	283.019	0.420669	1.28	up	0.046	C01172
d-Allose 6-phosphate	C6H13O9P	ESI+	283.019	0.420669	1.28	up	0.046	C02962
N-Acetylneuraminate 9-phosphate	C11H20NO12P	ESI+	428.037	3.966776	1.57	up	0.000	C06241
Cytosine	C4H5N3O	ESI+	112.0506	0.697346	1.76	up	0.002	C00380
d-Glyceraldehyde	C3H6O3	ESI-	135.0307	9.084401	1.59	up	0.046	C00577
d-Fructose 6-phosphate	C6H13O9P	ESI+	283.019	0.420669	1.28	up	0.046	C00085

## Data Availability

No data reported.

## Supplementary Materials

The following are available online, Figure S1: Fluorescently labeled PS-MPs of 1 µm diameter did not accumulate in liver of rare minnow, Figure S2: Multivariate statistical analysis in control and MPs exposure groups, Figure S3: KEGG pathway classification of the differential metabolites in “control vs. exposure”, Figure S4: The images for 1 μm fluorescent PS-MPs detected using a scanning electron microscope (SEM) and fluorescence microscopy, Table S1: Pathway analysis (KEGG) for metabolites and genes significantly affected by MP exposure, Table S2: Pathway analysis (KEGG) for metabolites and genes significantly affected by MP exposure.
