# Supplementary material for: Microplastics (Polystyrene) Exposure Induces Metabolic Changes in the Liver of Rare Minnow (Gobiocypris rarus)
Source: Molecules. 2022 Jan 18;27(3):584. doi: 10.3390/molecules27030584 (PMC8840292; doi:10.3390/molecules27030584)
Supplement: Supplementary file 1 [file molecules-27-00584-s001.zip › molecules-1523977-supplementary.pdf]

**Supporting Information**

# Microplastics (Polystyrene) Exposure Induce Metabolic Changes in the Liver of Rare Minnow (*Gobiocypris rarus*)

**Chunling Wang [1], Miaomiao Hou [1,2], Kunyu Shang [3], Huanshan Wang [1] and Jianwei Wang [1,*]**

[1] The Key Laboratory of Aquatic Biodiversity and Conservation of Chinese Academy of Sciences, Institute of Hydrobiology, Chinese Academy of Sciences, Wuhan 430072, China; chwang@ihb.ac.cn (C.W.); houmiao006@163.com (M.H.); hswang@ihb.ac.cn (H.W.)

[2] University of Chinese Academy of Sciences, Beijing 100049, China

[3] College of Fisheries,Huazhong Agricultural University, Wuhan, 430070, China; 15127315124@163.com (K.S.)

* Correspondence: wangjw@ihb.ac.cn (J.W.)

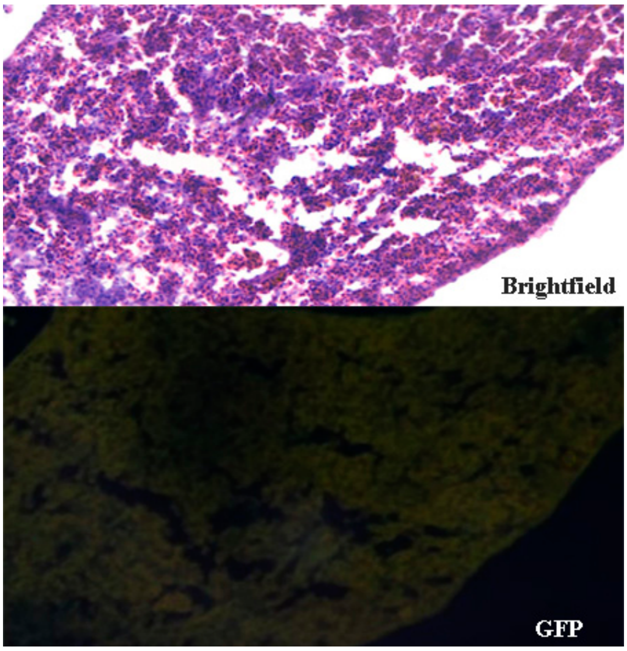


Figure S1. Fluorescently labeled PS-MPs of 1-μm diameter didn't accumulate in liver of rare minnow.

**Table S1.** Pathway analysis (KEGG) for metabolites and genes significantly affected by MP exposure

| Pathway | Description | Metabolites/Genes |
|---|---|---|
| ko00051 | Fructose and mannose metabolism | C00577,C00644,C05345,akr1b,sord,aldo,hk,tigar,pfkfb1,pfkfb4,tpi,pmm,gmpp,fcl,fbp ,pfka,fpgt,fuk |
| ko00360 | Phenylalanine metabolism | C03519,C05620,tat,ddc,mao,hpd,aldh3 |
| ko00040 | Pentose and glucuronate interconversions | C00817,C00514,glcak,akr1a1,kl,ugp2,akr1b,dhdh,sord,xylb |
| ko00760 | Nicotinate and nicotinamide metabolism | C16159,C16390,nadx,aox,puna,sirt3,sirt6,sirt7,nrk1,nrk2,nmnat,enpp1,enpp3,nadx |
| ko00052 | Galactose metabolism | C01216,C00577,ugp2,ganc,mgam,malz,galt,glb1,pgm2,g6pc,gla,hk,pfka,akr1b |
| ko00030 | Pentose phosphate pathway | C05345,C00577,gnl,h6pd,tkta,fbp,pfka,aldo,tala,pgm2,prps, |
| ko00561 | Glycerolipid metabolism | C00577,aldh,akr1a1,akr1b,glpk,gpat1,gpat2,cel,lipg,plpp1,plpp2,plpp3,lpin,lpl,dgk,d gat1,dgat2,gla |
| ko01200 | Carbon metabolism | C00577,C00085,C05345 |
| ko00280 | Valine, leucine and isoleucine degradation | C06000,mccc1,auh,hibch,hmgcs2,oxct,aacs,aldh,aox |
| ko00600 | Sphingolipid metabolism | C03640,plpp1,plpp2,plpp3,acer1,acer2degs,sgms,b4galt6,cerk,gla,smpd1,smpd4,enp p7,psap, |
| ko02010 | ABC transporters | C20570,abca5,abcb9,abcb11,abcc10,abcd2,abcd3,abcg2,abcg5,abcg8, |
| ko00240 | Pyrimidine metabolism | C00380,comeb,cdd,udp,dctpp1,dck,udk,entpd5,entpd6,dhodh,cad,psug,psuk,puna,c mpk1,enpp1,enpp3,dpys,deoa,tdk,ndk,rrm1,thya,dut,nt5c |
| ko00010 | Glycolysis / Gluconeogenesis | C05345, pgm,g6pc,hk,fbp,pfka,aldo,tpi,gapdh,eno,acee,akr1a1,aldh3,ldh,aldh,acss |
| ko00520 | Amino sugar and nucleotide sugar metabolism | C06241,C05345,cyb5r,uap1,hk,nagb,chit1,glcak,pgm,ga1t,uap2,fuk,pgm2,pmm,tsta3 |

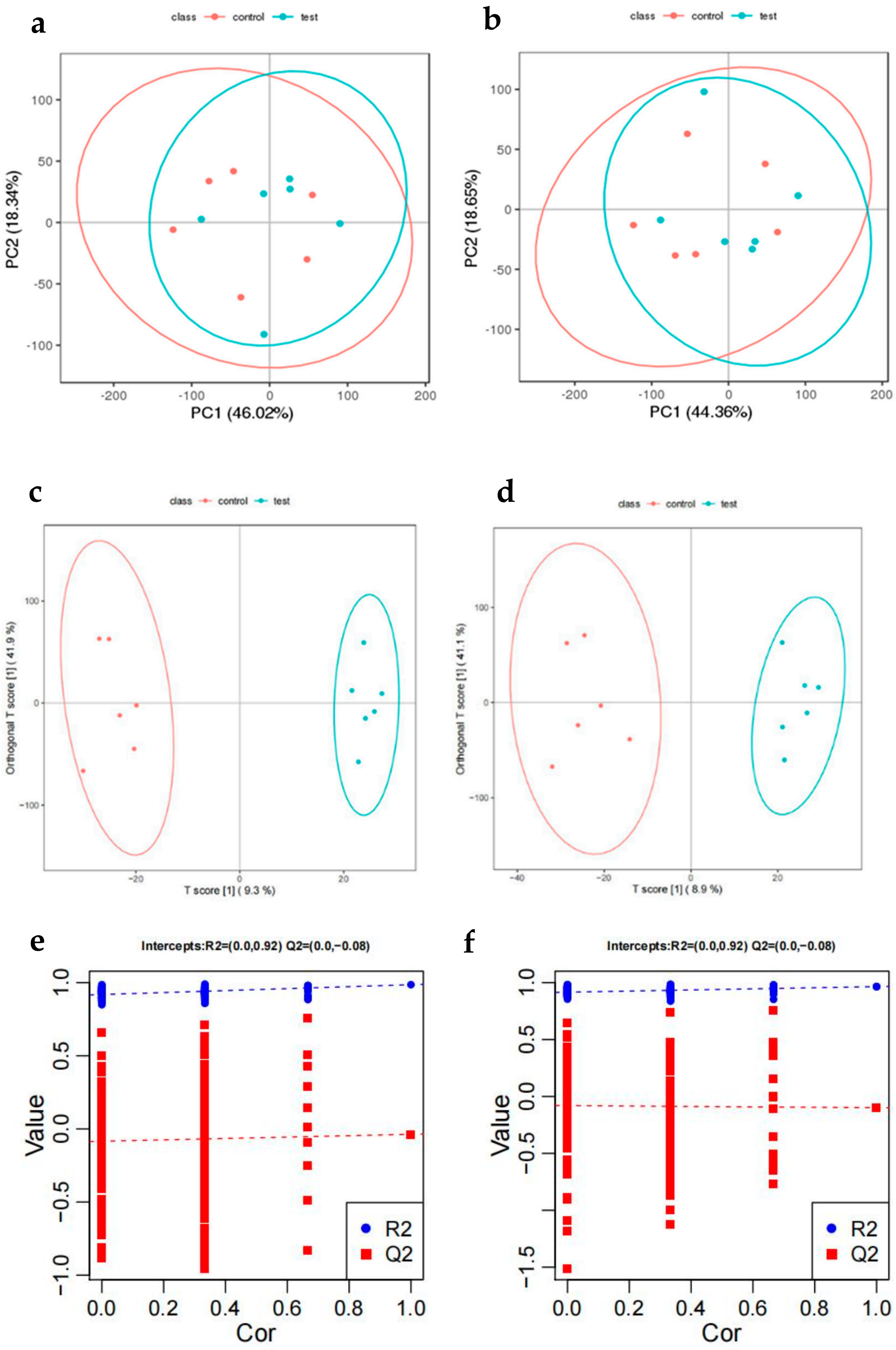


**Figure S2.** Multivariate statistical analysis in control and MPs-exposure groups. (a) Score scatter plot of PCA model of ESI+. (b) Score scatter plot of PCA model of ESI-. (c) OPLS-DA scores plot of ESI+. (d) OPLS-DA scores plot of ESI-. R2X=0.56, R2Y=0.991, Q2Y=0.689. (e) Verification diagram for OPLS-DA modle of ESI+. (f) Verification diagram for OPLS-DA modle of ESI-.

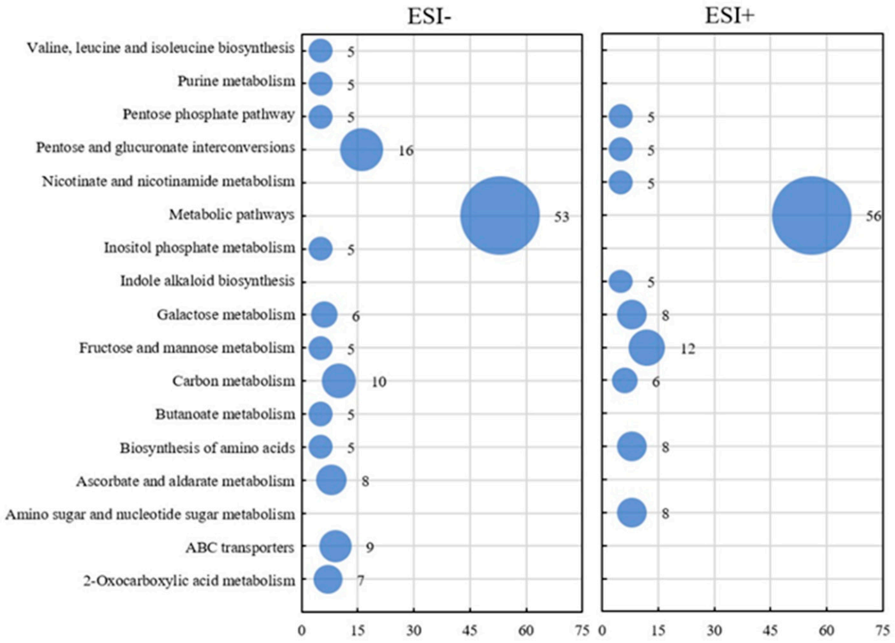


**Figure S3.** Kyoto Encyclopedia of Genes and Genomes (KEGG) pathway classification of the differential metabolites in "control vs exposure".

**A** **B**

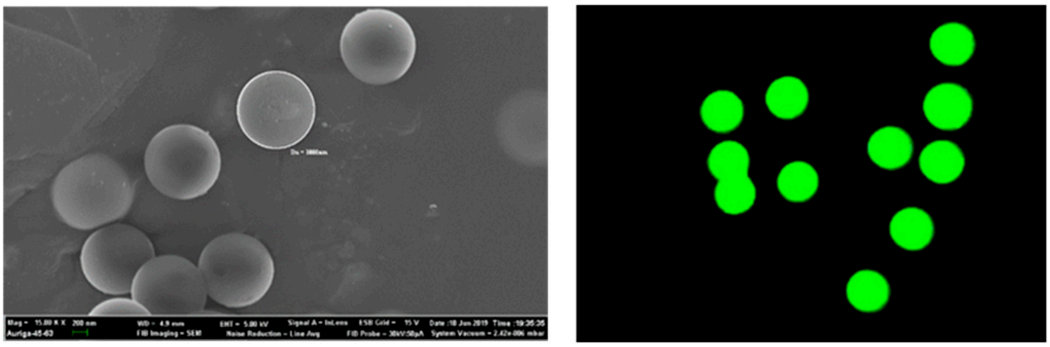

**Figure S4**. The images for 1 μm fluorescent PS-MPs detected by (A) scanning electron microscope (SEM) and (B) fluorescence microscopy.

**Table S2.** Primers used in the present study

| Name of primer | Sequence (5'-3') |
|---|---|
| FASN-F | AACTGTAGACCTGTAACGGGAG |
| FASN-R | AACAACACTTCATTCTGGCAA |
| ACSF2-F | GGGGATACTGCGTGATGCTG |
| ACSF2-R | CCTTTATGCGTCCTTCTATTCTG |
| FAXDC2-F | AGACACATTCAACATTTCTCACAT |
| FAXDC2-R | GCGACCTTTTACACCACAGA |
| ELOVL5-F | GCGTGTCTGAAGTGAAGTGC |
| ELOVL5-R | GTGTCGTCAATGTGAAAGCG |
| CPT1A-F | TCGTCTACAAGAACGGTAAAATG |
| CPT1A-R | CTGTAGTCTGGTAGGGGGCG |
| β-actin-F | GTCCGTGACATCAAAGAG |
| β-actin-R | ACCGCAAGATTCCATAC |
